# Supplementary material for: Mode of intracontinental mountain building controlled by lower crustal composition and mantle lithosphere depletion
Source: Nat Commun. 2025 Oct 24;16:9404. doi: 10.1038/s41467-025-63468-1 (PMC12552677; doi:10.1038/s41467-025-63468-1)
Supplement: Supplementary file 1 — Supplementary Information [file 41467_2025_63468_MOESM1_ESM.pdf]

Supplementary Information for

Mode of intracontinental mountain building controlled by lower crustal composition and mantle lithosphere depletion

Xi Xu, Andrew V. Zuza, Taras Gerya, Lin Chen, Xingtao Kuang,

Hanlin Chen, Baodi Wang, Jingao Liu, Xuhua Shi,

Yanyun Sun, Lei Wu, Song Han, Xiubin Lin,

Shufeng Yang and An Yin

This PDF file includes:

Figs. S1 to S16

Tables S1 to S2

Fig. S1

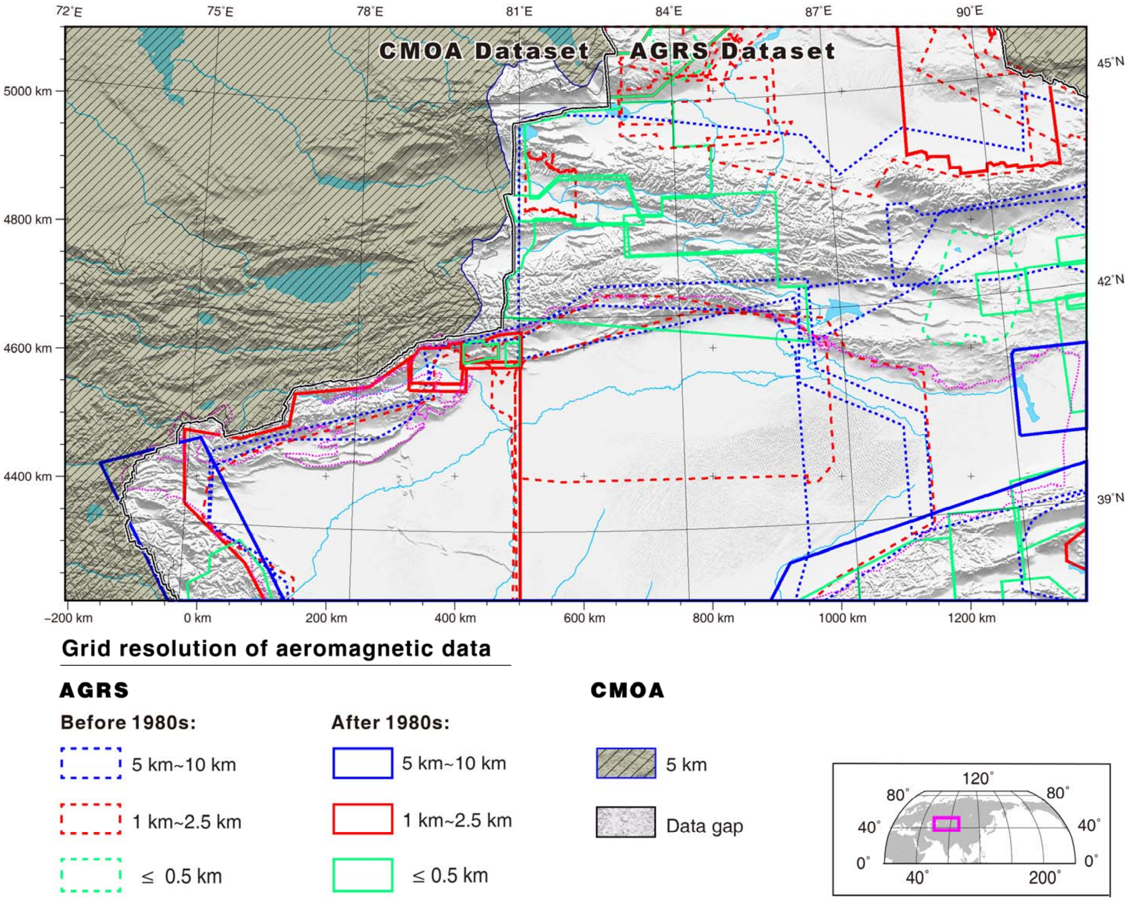

Fig. S1 AGRS and CMOA aeromagnetic datasets in and around the Tian Shan. Colored polygons show different flight-line spacing for compiled data. The two digital data grids are stitched together along the blue line. The aeromagnetic compilation for this study region combines more than 20 aeromagnetic surveys, flown between 1960s and 2000s and varied in flight-line spacing of 0.5/1/2.5/5/10 km and elevation ranging from 0.6 to 3 km following the terrain. The non-colored regions indicate the grid gaps. AGRS, China Aero Geophysical Survey and Remote Sensing Center for Natural Resources, China Geological Survey; CMOA, China Mainland, Offshore and Adjacent areas. Data sources: China Geological Survey Geocloud Database (http://geocloud.cgs.gov.cn/) and refs[1-4,5].

Fig. S2

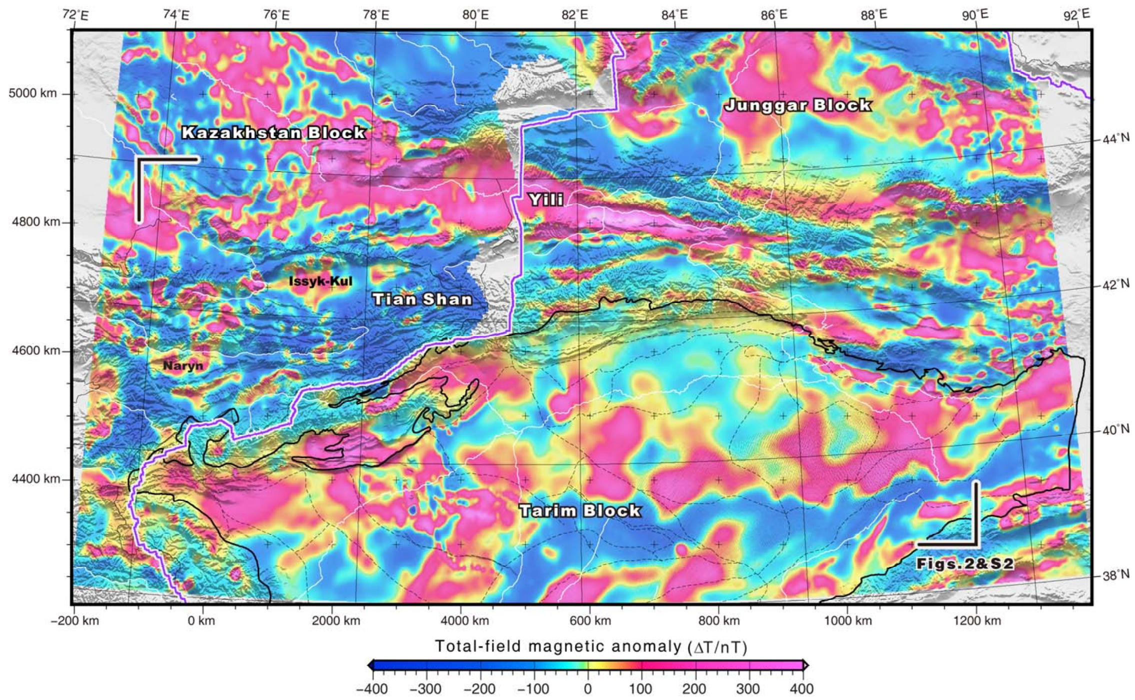

Fig. S2 Aeromagnetic data sets of AGRS and CMOA within and around Tian Shan, overlain on hillshade digital elevation model (https://www.ngdc.noaa.gov/mgg/global/). The two digital data grids are stitched together along the white-violet line with the reduction-to-pole (RTP) correction. The warm red and cool blue colors depict magnetic highs and lows, respectively. Gray uncolored polygon regions are magnetic data gaps. The high-resolution aeromagnetic data set with a 5 km × 5 km grid could be used for research purposes via the application in the China Geological Survey Geocloud Database (http://geocloud.cgs.gov.cn/), or digitized from the high-resolution aeromagnetic map of the China mainland[5, 6] and adjacent areas[7].

Fig. S3

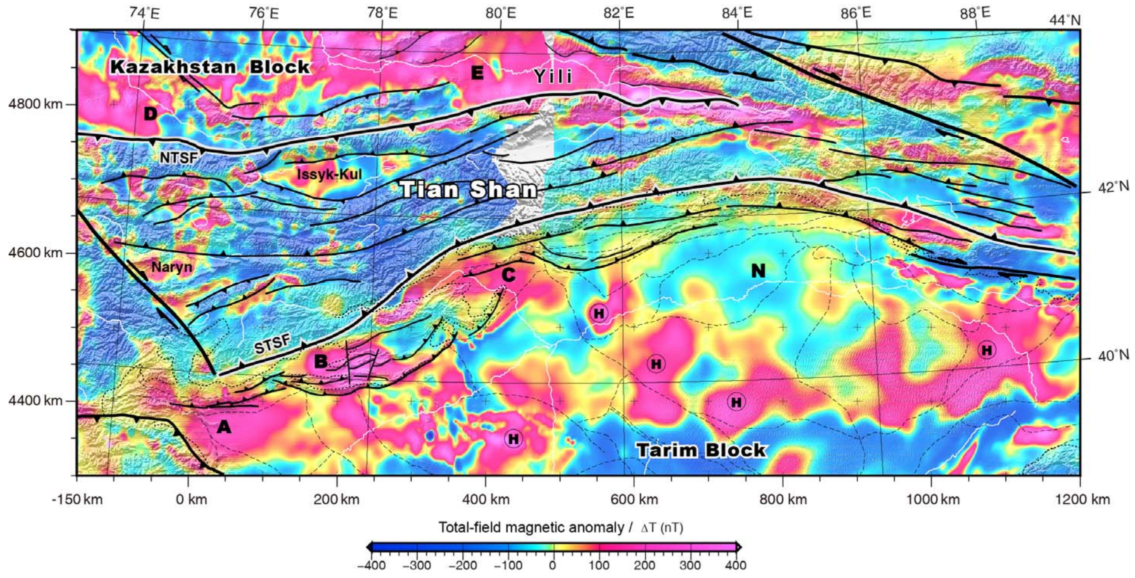

Fig. S3 Magnetic and tectonic map of the Tian Shan and its surrounding regions. Composite magnetic map of the Tian Shan and its bounding Tarim-Kazakhstan block showing total-field aeromagnetic data overlain on hillshade digital elevation model, corrected by the reduction to pole. Labels A-E and circled-H anomalies represent the magnetic highs (high amplitude). The gray-black lines represent the locations of southern and northern margin of Tian Shan, South Tian Shan Fault (STSF) and North Tian Shan Fault (NTSF).

Fig. S4

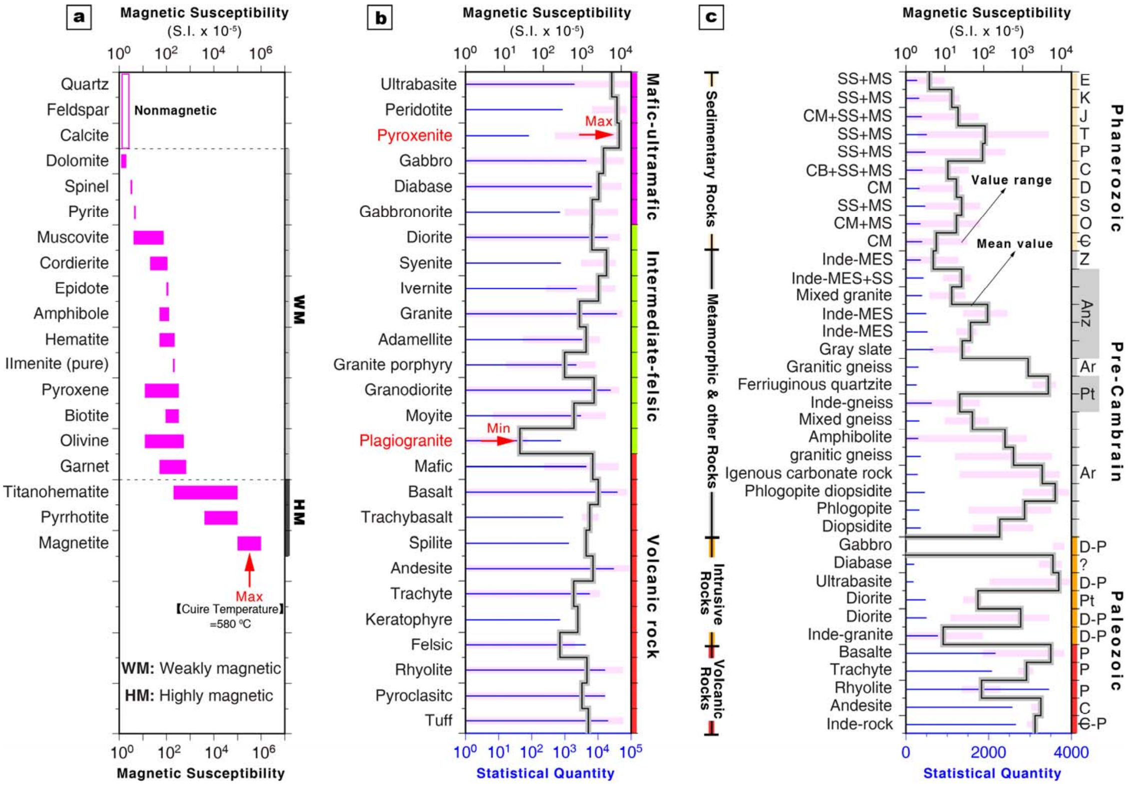

Fig. S4 (a) Magnetic susceptibility of common rock-forming minerals[8, 9]; HM, highly magnetic; WM, weakly magnetic. **(c)** Statistics of magnetic susceptibility of various rocks across the China mainland, totalling 248,756 rock samples in the field[5]. **(c)** Statistics of 25,643 detailed magnetic susceptibility measurements from surface exposure and drilled-core rocks including sedimentary, metamorphic, intrusive, volcanic and other rocks within and around the Tian Shan-Tarim basin[5]. SS, Sandstone; MS, Mudstone; CM, Conglomerate; CB, Carbonatite; Inde-MES, indefinite metamorphic rock; Inde-gneiss, indefinite gneiss; Inde-granite, indefinite granite; Inde-rock, indefinite intrusive rock.

Fig. S5

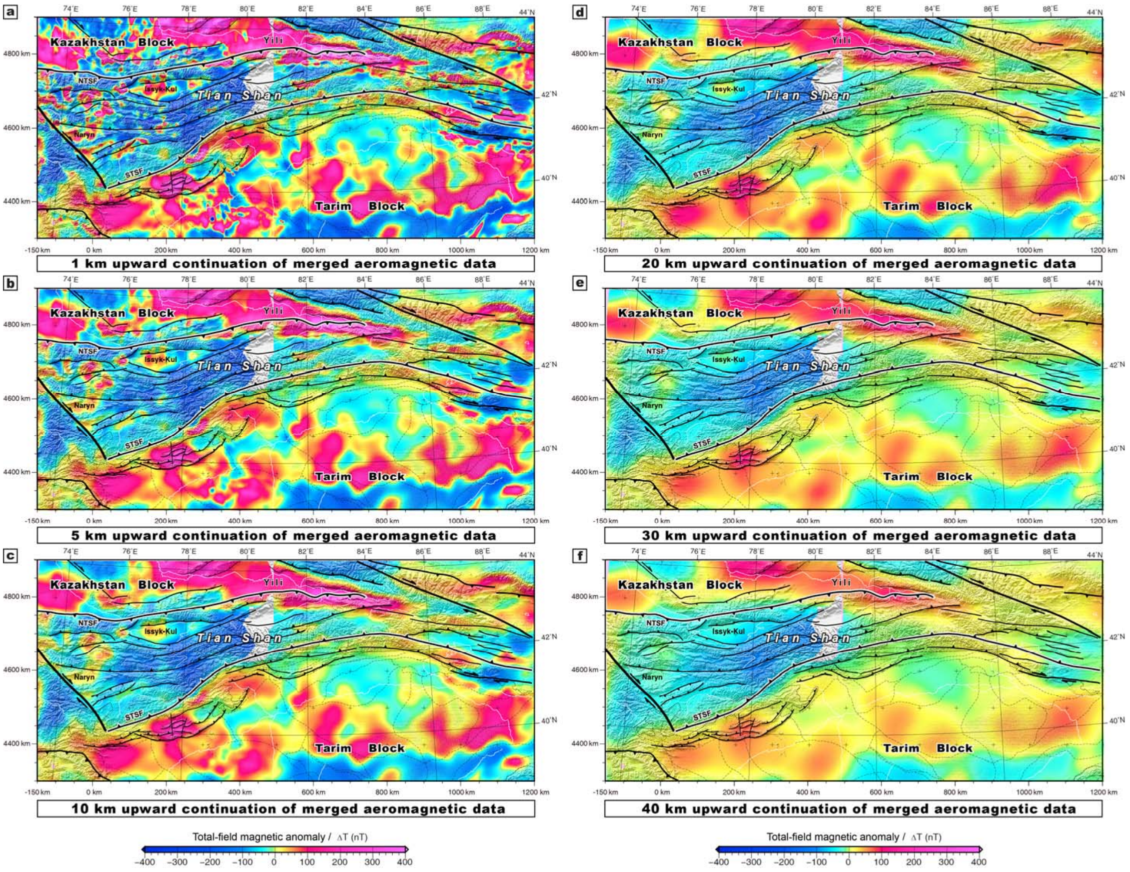

Fig. S5 Map of magnetic anomalies at different upward continuation values to enhance regional and deep magnetic features. Panels **a** through **f** correspond to upward continuation of 1 km, 5 km, 10 km, 20 km, 30 km and 40 km, respectively. All magnetic maps are overlain on hillshade digital elevation model.

Fig. S6

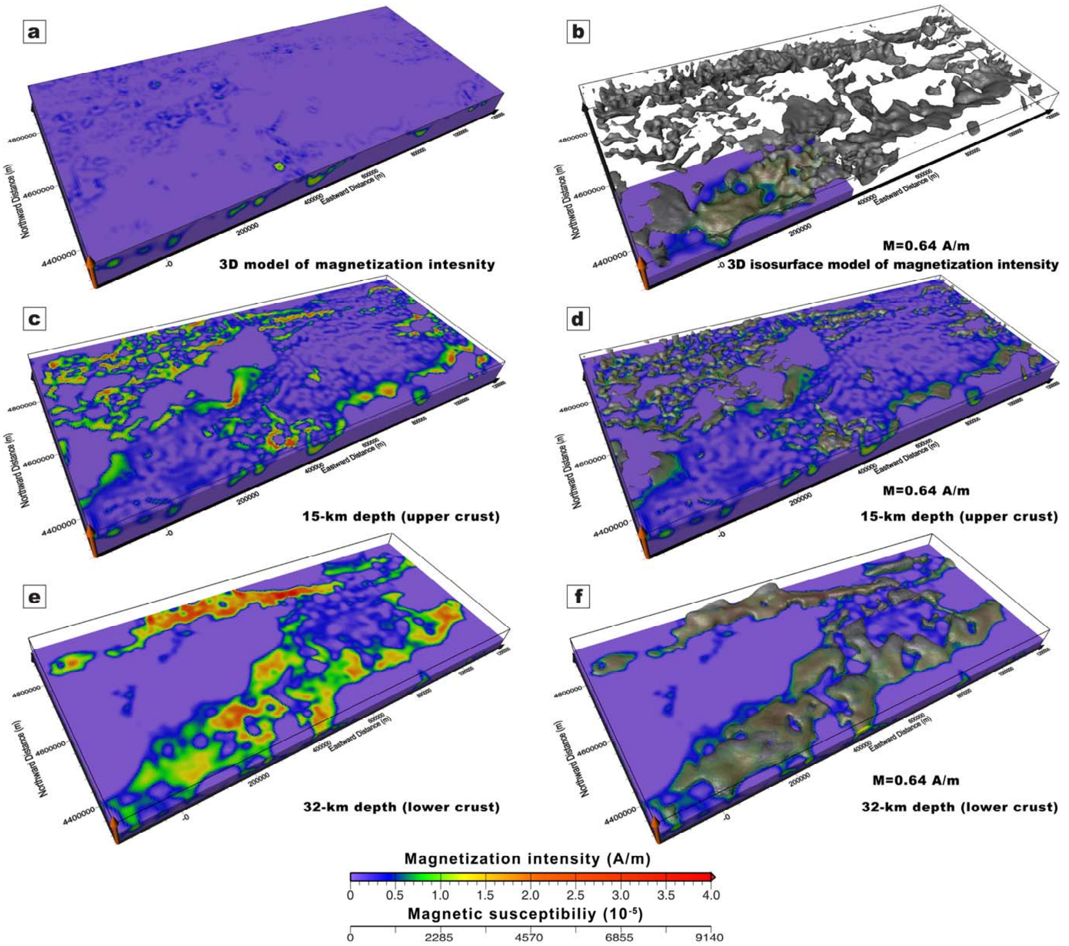

Fig. S6 Crust-scale magnetization intensity model for the Tian Shan and its bounding blocks. **(a)**, Three dimensional (3D) visualization is based on the regularization inversion model with 271 × 121 × 60 grid cells at a resolution of 5 km × 5 km × 1 km; **(b)**, 3D view of iso-surface magnetization model (magnetization intensity is 0.4 A/m); **(c)**, 15-km upper crustal-scale horizontal slice of the inverted magnetization intensity model, overlain with 3D top-view transparent iso-surface magnetization model **(d)**; **(e)**, 32-km lower crustal-scale horizontal slice of the inverted magnetization intensity model, overlain with 3D bottom-view transparent iso-surface magnetization model **(f)**. The volume magnetic susceptibility (k) was calculated from k=Mμ_0/ΔT [10], where M is the induced magnetization intensity, μ_0 is the magnetic permeability of free space ($\mu_0=4\pi\times10^{-7}$ H/m), and ΔT is the total geomagnetic intensity of Tian Shan and its surrounding domains (ΔT=55000 nT).

Fig. S7

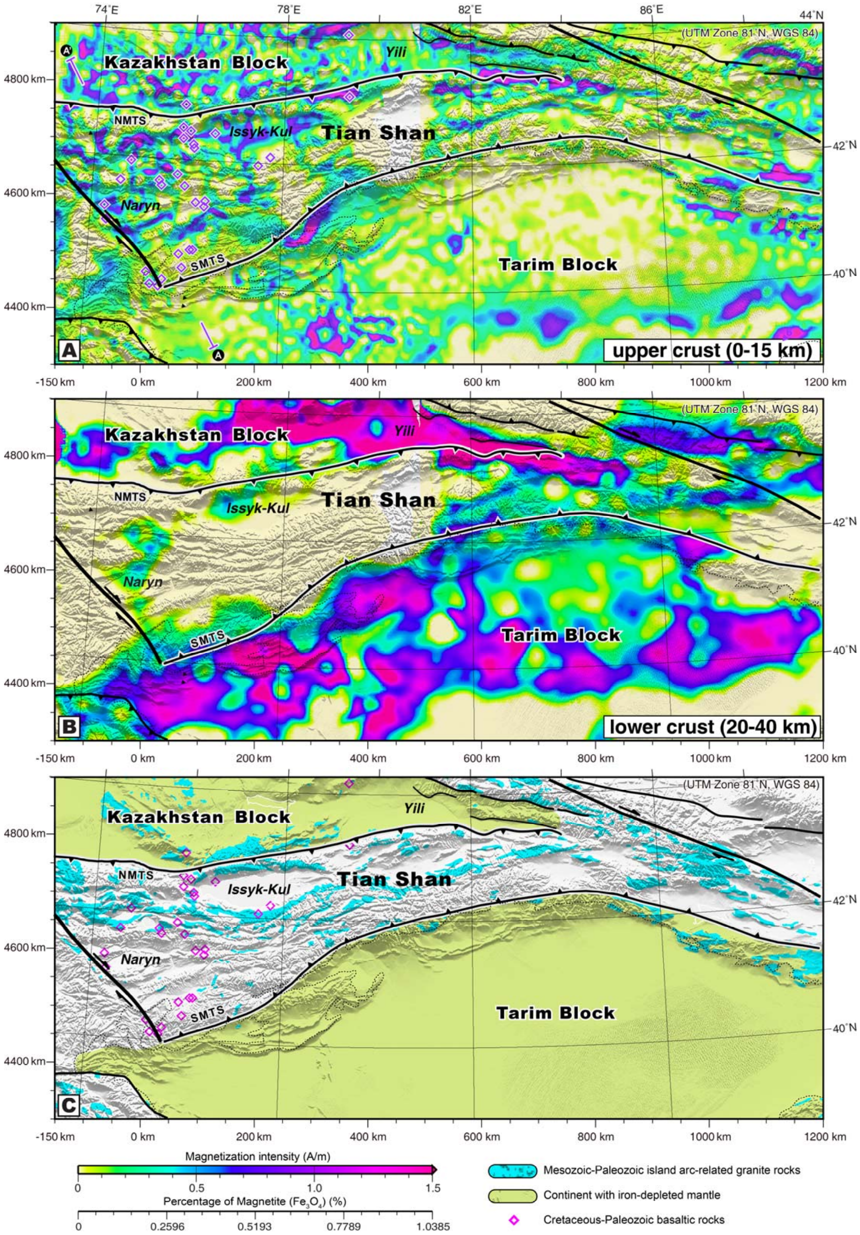

Fig. S7 Magnetization intensity map of the Tian Shan and its surrounding regions, calculated from the 3-D regularized inversion of total magnetic intensity. The maps of average magnetization intensity of upper crust **(a)** and lower crust **(b)**, averaged from each 1-km horizonal slice model, overlain on hillshade digital elevation model. The volume percentage of magnetite in the crust (Mt. %) is estimated from an empirical relationship (Methods). **(c)** Locations of Paleozoic and Mesozoic arc-related granite and Cretaceous-Paleozoic basaltic rocks in the Tian Shan[11] and the geological interpretation of the magnetization intensity.

Fig. S8

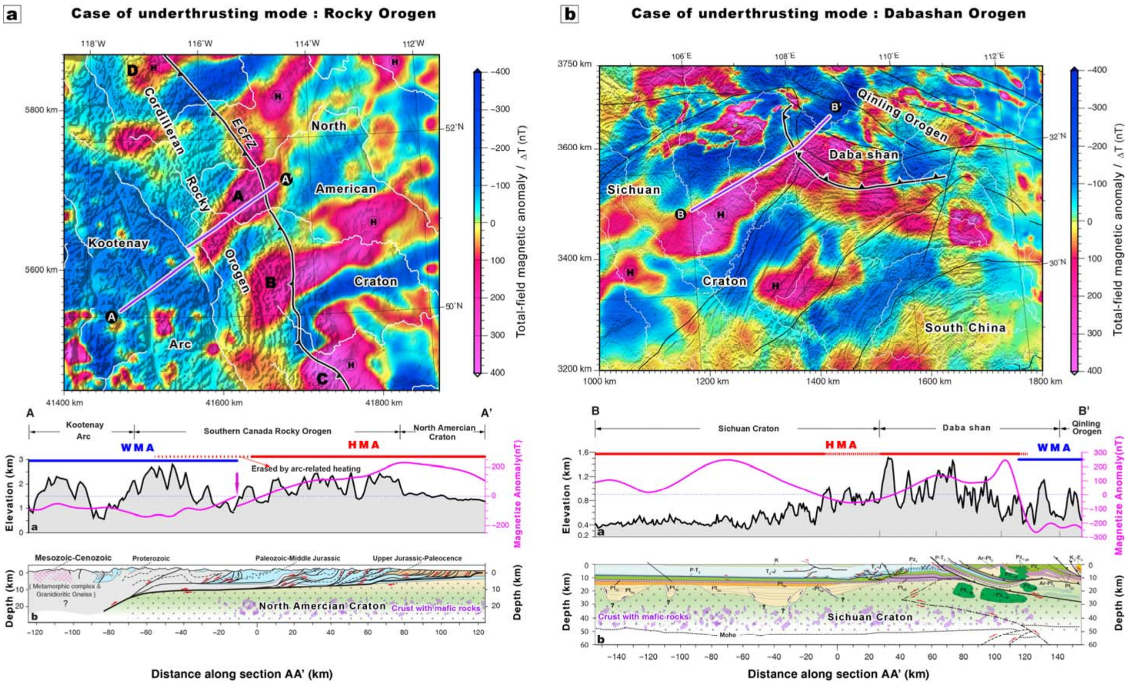

Fig. S8 Magnetic signals across the Canadian Rocky **(a)** and China Daba Shan **(b)** orogens. The geological profile A-A' in the Figure **(a)** and **(b)** is from Price[12] and Dong et al.[13], respectively. The magnetic data of Figure **(a)** and **(b)** is derived from EMAG2 V2 data set[14] and China Mainland aeromagnetic map[5, 6]. Topography and magnetic anomaly signal across the analyzed profile. HMA, highly positive magnetic anomaly. WMA, weakly negative magnetic anomaly.

Fig. S9

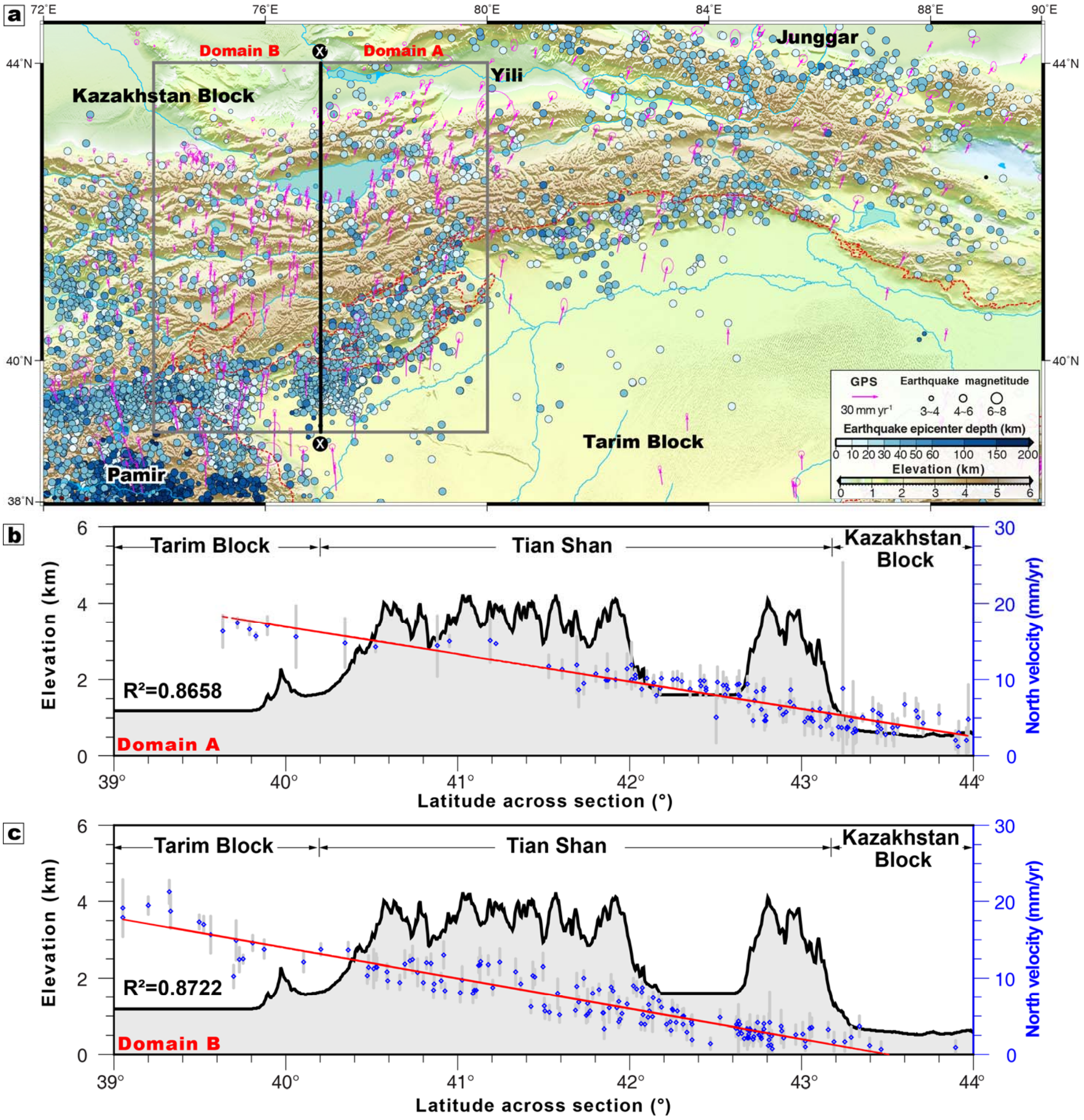

Fig. S9 (a). Seismicity from January 1964 to August 2020 across the Tianshan and its surrounding areas (earthquake magnitude >3), from the USGS (http://earthquake.usgs.gov/earthquakes/). Pink arrows are Global Positioning System (GPS) velocities[15]. Profile XX' is the projected section for northward component of GPS velocity across the domains A and B. Northward velocity components of GPS controlling points across the domains A and B projected onto profile XX' (location in Fig. a), corresponding to the sections **(b)** and **(c)**, respectively.

Fig. S10

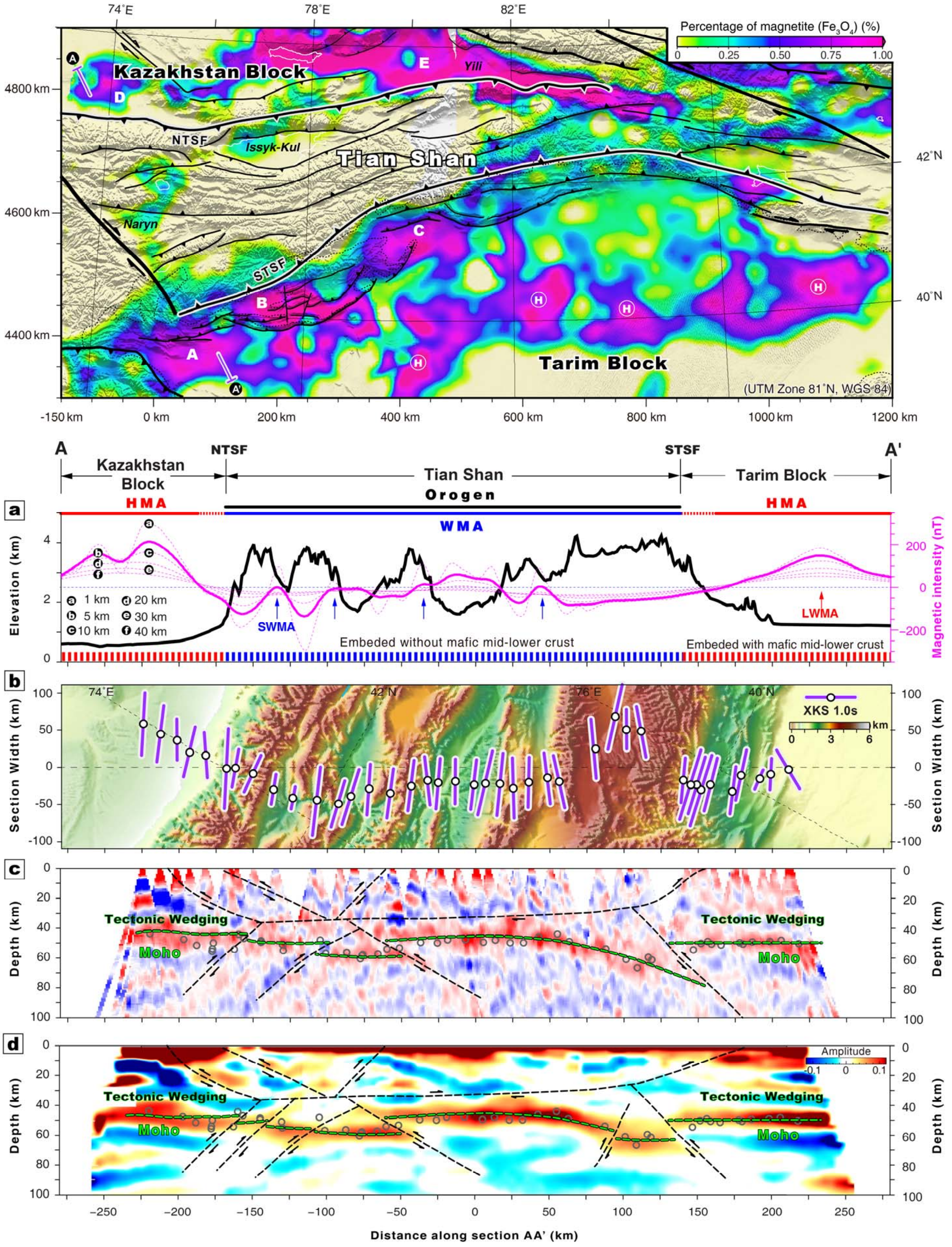

Fig. S10 (a), Magnetic anomaly and topography across the analyzed profile AA'. Red and blue arrows represent the locations of long-wavelength and short-wavelength magnetic

anomalies (LWMA and SWMA), respectively. The SWMA indicate upper crustal, magnetite-bearing igneous intrusions. Circled-a to f represent the altitude of upward continuation for the magnetic data (Fig. S3). The Tian Shan and its bounding blocks are indicated by highly and weakly magnetic anomalies (HMA and WMA), respectively. **(b)**, Apparent anisotropy of crust and upper mantle lithosphere across the section AA'[16]. **(c)** and **(d)**, Moho morphology imaged by Zhang et al.[17] via the common-conversion-point (CCP) stacking technique, and Li et al.[18] via the joint inversion, respectively. These are plotted with faults (dotted lines) and Moho discontinuities (dotted green thick lines), reinterpreted from both receiver function profiles, of which the structural architecture are non-unique structural models. We use Zhang et al.'s CCP profile and their H-k-c Moho picks (gray circles)[17], which can be more conservatively interpreted to show a single Moho surface that is offset by several thrust structures (~10 km or less offset). Note both images provide similarly consistent interpretations.

Fig. S11

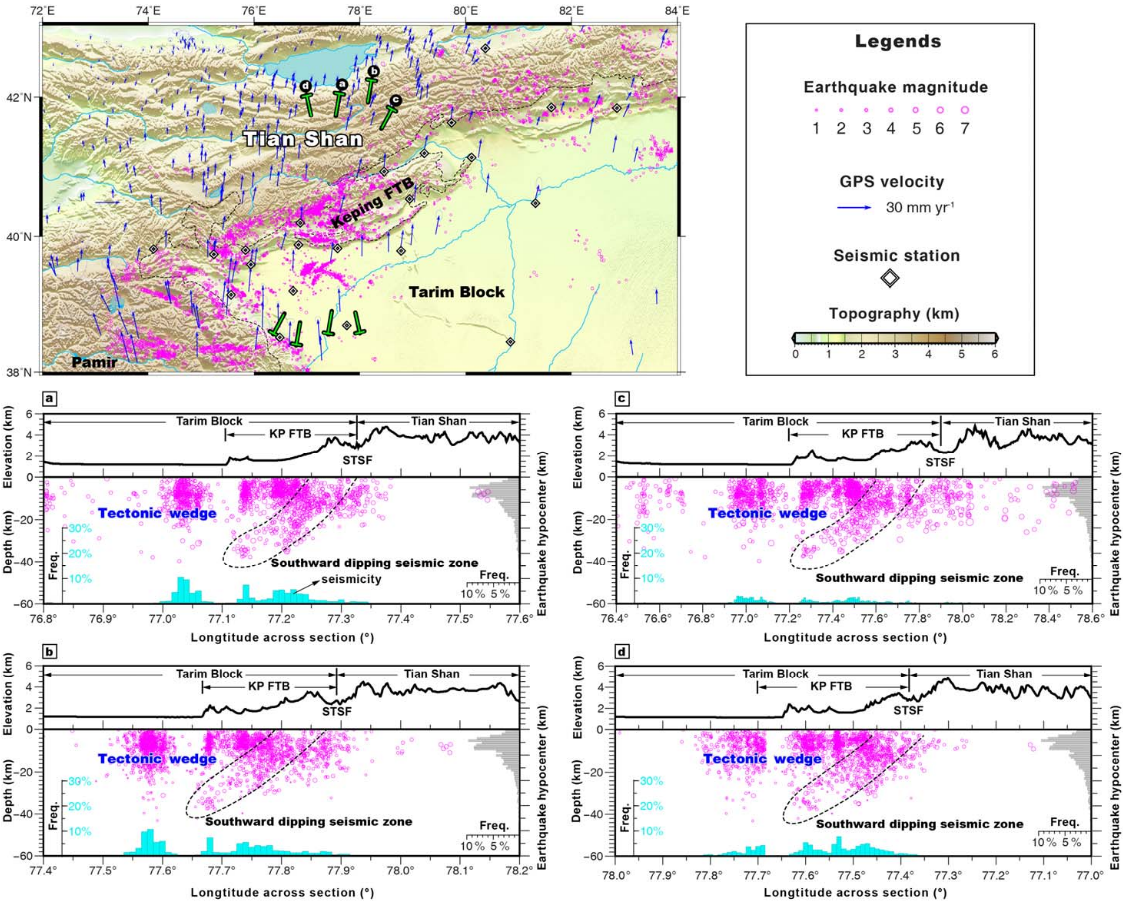

Fig. S11 Relocated epicenter distribution across the Tarim block and Tian Shan and southward dipping seismic zone. The white-violet diamonds are seismic stations, and pink dots are relocated 43,484 earthquake epicenters from the China Earthquake Networks Center (https://www.cenc.ac.cn), which were recorded by 1697 seismic stations from 2009 to 2016. 17,504 epicenter-relocated earthquakes are produced by using double-difference location algorithm (https://www.cenc.ac.cn). Projected seismicity onto a, b, c and d profiles. Note that all profiles reveal a south-dipping cluster of seismicity at the boundary between the Tarim block and the Tian Shan. The southern Tian Shan thrust belt (Keping fold-and-thrust belt, KP FTB) is a south-directed, north-dipping thin-skinned thrust belt that overlies the observed south-dipping seismicity in these figures. This observation suggests wedge or "flake" tectonics, and this geometry guides are pure-shear cross-section model in Fig. 2.

Fig. S12.

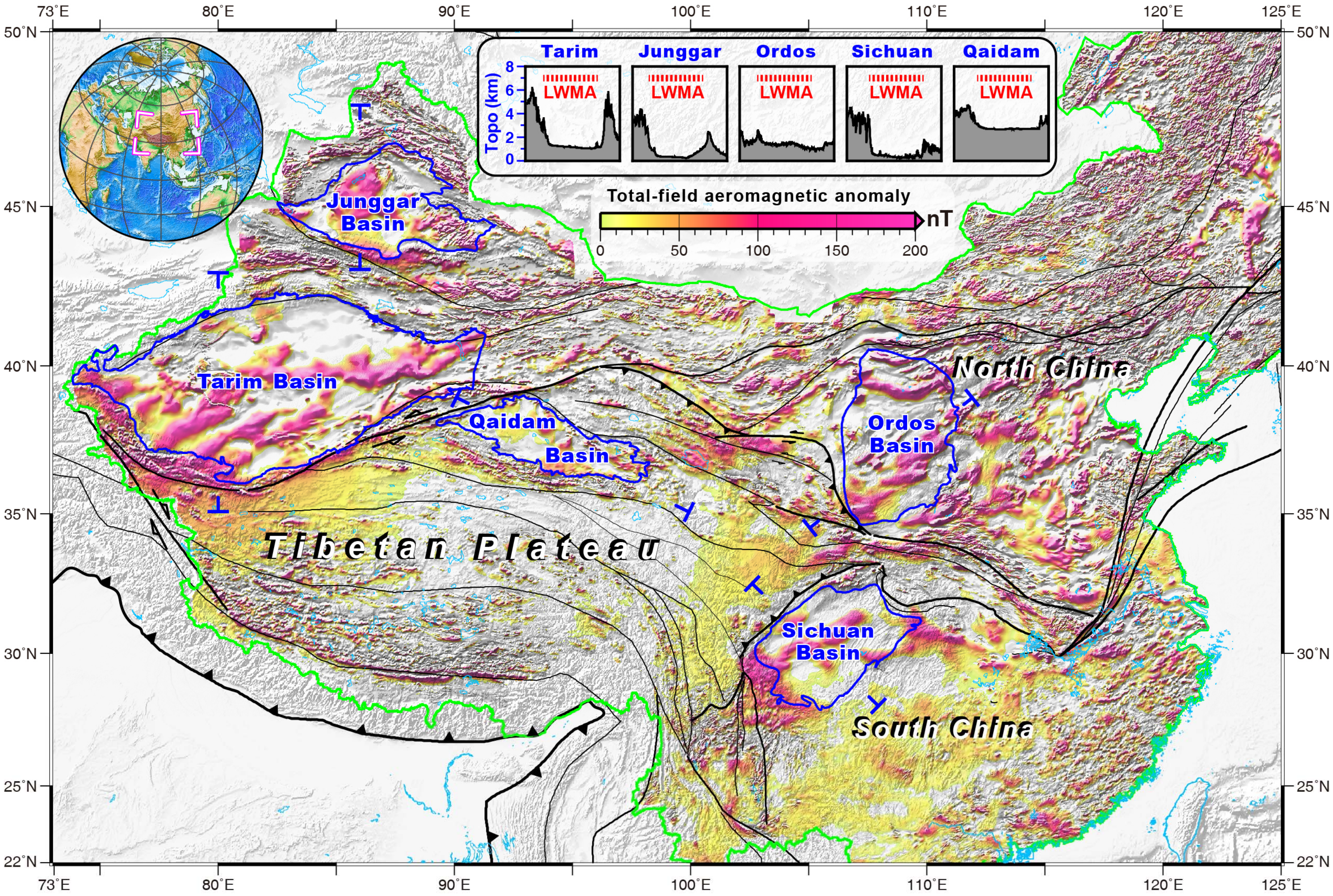

Fig. S12 Aeromagnetic imaging of the Chinese continental crust. The five low-relief stable cratonic blocks located around the Tibetan plateau, including Tarim, Sichuan, Ordos, Junggar and Qaidam, display a signal of long-wavelength magnetic anomalies (LWMA). Topographic profiles across these cratonic basins were derived from the digital elevation model. The total-field magnetic anomaly intensity (ΔT) map of China mainland[5, 6, 9] was released by China Aero-Geophysical Survey and Remote Sensing Center for Natural Resources (AGRS), China Geological Survey. Thick green and turquoise color lines represent the high-resolution aeromagnetic grid and lake-river-sea boundaries, respectively.

Fig. S13.

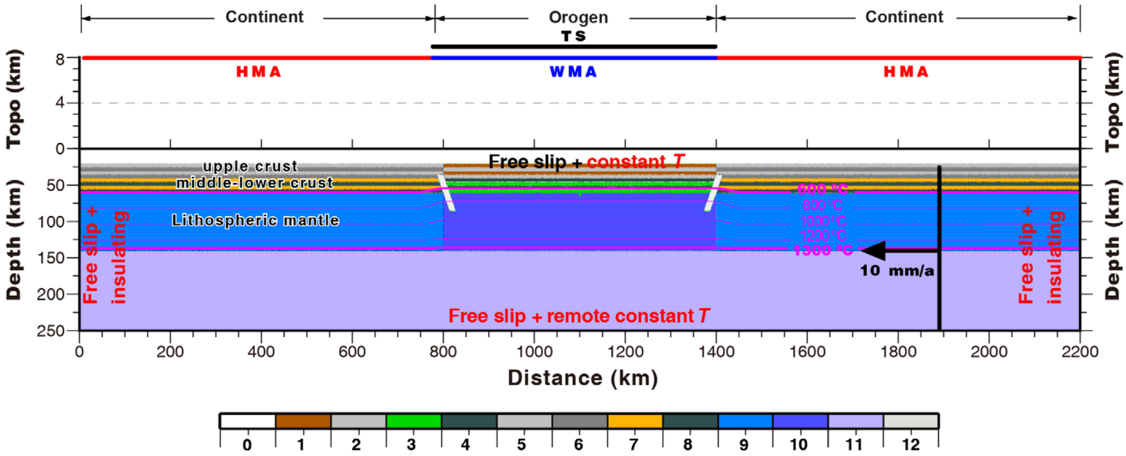

Fig. S13 Initial model configuration and thermal-mechanical boundary conditions. The black vertical bar with lateral arrow indicates the location where convergence rate is imposed. The composition codes are: 0, sticky air; 1 and 2, orogen upper crust; 3 and 4, orogen middle-lower crust; 5 and 6, continent upper crust; 7 and 8, continent middle-lower crust; 9, continent lithosphere mantle; 10, orogen lithosphere mantle; 11, asthenosphere; 12, pre-existing weak zone. TS, Tian Shan topographic range

Table S1

Table S1 Rheological parameters of material used in this study [a]

Material	Flow law	A_D (Pa^n s)	n	E_a (J/mol)	V (cm^3/mol)	sin(φ)	C (MPa)	ρ_0 (kg/m^3)	k (W/K/m)	H_r (μW/m^3)
Stick air	N.A.	1.00×10^{18}	1.0	0	0	0	0	1	200	0
Water	N.A.	1.00×10^{18}	1.0	0	0	0	0	1000	200	0
UCC	WQ	1.97×10^{17}	2.3	1.54×10^{5}	8	0.3-0.15	10-1	2700	0.64+807/(T+77)	1.8
SLM	WOL	5.01×10^{20}	4.0	4.70×10^{5}	8	0.4-0.2	10-1	3300	0.73+1293/(T+77)	0.022
CLC1	MG	1.13×10^{21}	4.2	4.45×10^{5}	8	0.3-0.15	10-1	2900	1.18+474/(T+77)	0.18
CLC2	FG	4.98×10^{20}	3.1	2.43×10^{5}	8	0.3-0.15	10-1	2900	1.18+474/(T+77)	0.18
CLM1	DOL	3.98×10^{16}	3.5	5.32×10^{5}	12	0.6-0.3	10-1	varied	0.73+1293/(T+77)	0.022
CLM2	DOL×0.25	3.11×10^{14}	3.5	5.32×10^{5}	12	0.6-0.3	10-1	varied	0.73+1293/(T+77)	0.022
OLC1	FG	4.98×10^{20}	3.1	2.43×10^{5}	8	0.3-0.15	10-1	2900	1.18+474/(T+77)	0.18
OLC2	MG	1.13×10^{21}	4.2	4.45×10^{5}	8	0.3-0.15	10-1	2900	1.18+474/(T+77)	0.18
OLM	DOL×0.25	3.11×10^{14}	3.5	5.32×10^{5}	12	0.6-0.3	10-1	3300	0.73+1293/(T+77)	0.022
CWZ	WQ	1.97×10^{17}	2.3	1.54×10^{5}	0	0	1	3000	1.18+474/(T+77)	0.18
Seed	WQ	1.97×10^{17}	2.3	1.54×10^{5}	0	0	1	3000	1.18+474/(T+77)	0.18

[a] A_D is the pre-exponential constant; n is the stress exponent; E_a is the activation energy; V is the activation volume; φ is the friction angle; C is the cohesion. For material: UCC, upper continental crust; SLM, sub-lithospheric mantle; CLC1, continental lithospheric crust (iron-depleted); CLC2, continental lithospheric crust (iron-rich); CLM1, continental lithospheric mantle (iron-depleted); CLM2, continental lithospheric mantle (iron-rich); OLC1, orogenic lower crust (felsic); OLC2, orogenic lower crust (mafic); OLM, orogenic lithospheric mantle; CWZ, crustal weak zone. For flow law: N.A., not applicable; WQ, wet quartz; WOL, wet olivine; MG, mafic granulite; FG, felsic granulite; DOL, dry olivine. All the other parameters follow Tables in the references[19, 20].

Table S2

Table S2 Parameters and results of conducted numerical experiments

Model name	Orogen Moho temperature (℃)	Continental Moho temperature (℃)	Orogen lower crust flow law	Continental lower crust flow law	Depletion density [a] (kg/m^3)	Deformation Mode
TSM01	700	600	Felsic granulite	Mafic granulite	30	Pure shear
TSM02	600	600	Felsic granulite	Mafic granulite	30	Simple shear
TSM03	600	600	Felsic granulite	Felsic granulite	30	Pure shear
TSM04	600	600	Mafic granulite	Mafic granulite	30	Simple shear
TSM05	700	600	Felsic granulite	Mafic granulite	0	Simple shear
TSM06	600	600	Felsic granulite	Mafic granulite	0	Simple shear
TSM07	600	600	Felsic granulite	Felsic granulite	0	Pure Shear
TSM08	600	600	Mafic granulite	Mafic granulite	0	Simple shear
TSM09	600	600	Felsic granulite	Mafic granulite	60	Pure Shear
TSM10	600	600	Felsic granulite	Felsic granulite	60	Pure Shear
TSM11	600	600	Mafic granulite	Mafic granulite	60	Simple shear
TSM12	600	600	Felsic granulite	Mafic granulite	-30	Simple shear
TSM13	600	600	Felsic granulite	Felsic granulite	-30	Pure Shear
TSM14	600	600	Mafic granulite	Mafic granulite	-30	Simple shear
TSM15	600	600	Felsic granulite	Mafic granulite	-60	Simple shear
TSM16	600	600	Felsic granulite	Felsic granulite	-60	Pure Shear
TSM17	600	600	Mafic granulite	Mafic granulite	-60	Simple shear

[a] A negative depletion density means that the lithospheric mantle of the continent is denser than that of the orogen.

Fig. S14.

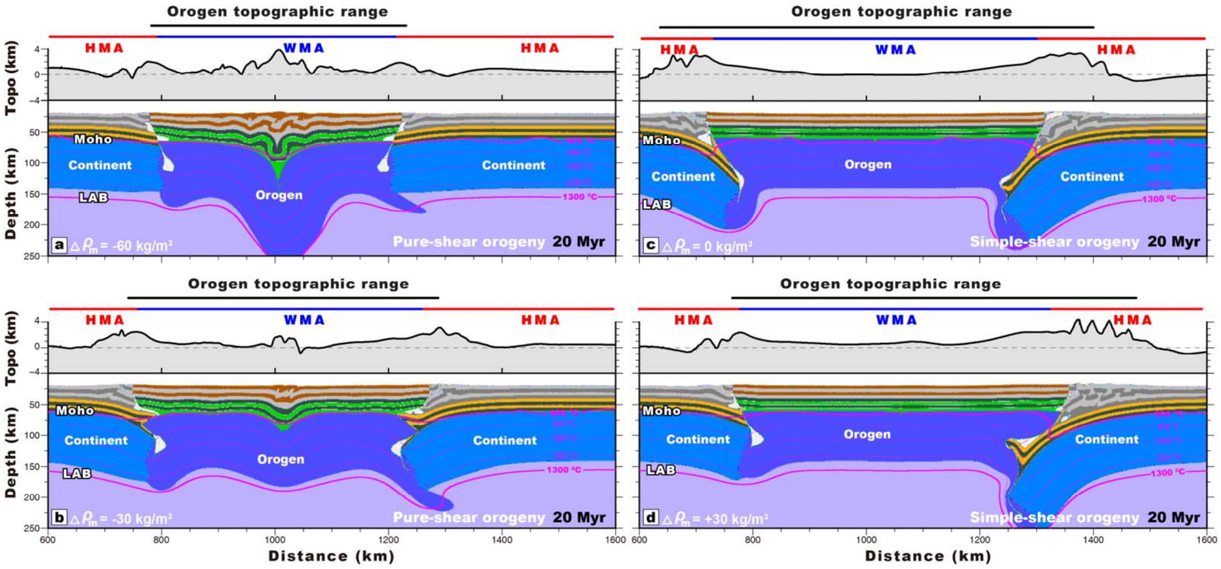

Fig. S14 Geodynamic modeling of the role of lithospheric mantle depletion-induced density contrast on the orogeny style. Collision between two continents with iron-fertile and iron-depleted mantle density varying from △ρ=+30 to -60 kg/m^3. The models with lithospheric mantle depletion-induced density contrast of -60 kg/m^3 **(a)** and -30 kg/m^3 **(b)** lead to pure shear-style thickening of an orogen via tectonic wedging, whereas 0 kg/m^3 **(c)** and +30 kg/m^3 **(d)** lead to underthrusting / subduction of continental lithosphere under the orogen. Here, the density is expressed as $\Delta\rho_m = \rho_{OLM} - \rho_{CLM}$, where ρ_{OLM} and ρ_{CLM} represent the reference density of the orogen and continent lithospheric mantle, respectively. The lower crusts of the continental and orogenic lithosphere are equipped with mafic and felsic rocks, respectively. The simulation results are presented at time of 20 Myr after model initiation. The initial thermal structure of the orogen is same to its bounding continent. The initial temperature increases linearly from 0 °C at the model surface to 600 °C at the crust base, and continues to increase to 1330 °C at the lithosphere base. Convergence rate is 10 mm/yr. Other parameters contrast between the orogen and its bounding continents are presented in Tables S1 and S2.

Fig. S15.

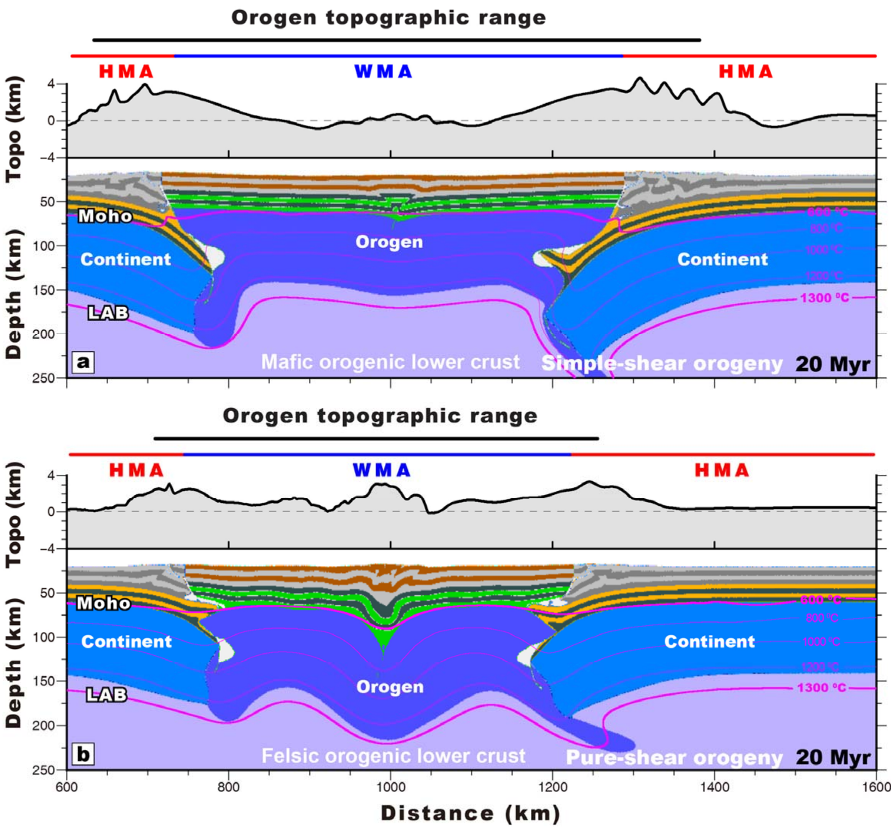

Fig. S15 Geodynamic modeling of the role of lower crustal composition on the orogeny style. Collision between two continents with mafic **(a)** and felsic **(b)** orogenic lower crust, leading to respectively underthrusting / subduction of continental lithosphere under the orogen **(a)** and pure shear-style thickening of an orogen via tectonic wedging **(b)**. Orogen-bounding continents are here equiped with mafic lower crust. The models are equipped with lithospheric mantle depletion density of -30 kg/m^3. The simulation results are presented at time of 20 Myr after model initiation. The initial thermal structure of the orogen is same to its bounding continent. The initial temperature increases linearly from 0 °C at the model surface to 600 °C at the crust base, and continues to increase to 1330 °C at the lithosphere base. Convergence rate is 10 mm/yr. Other parameters contrast between the orogen and its bounding continents are presented in Tables S1 and S2.

Fig. S16.

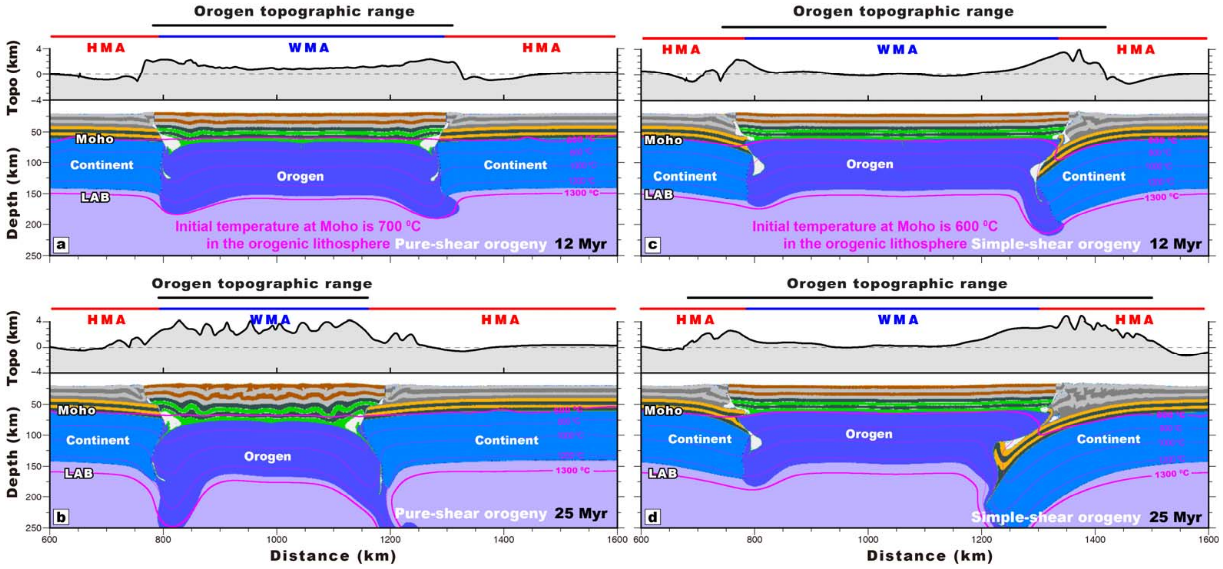

Fig. S16 Geodynamic modeling of the role of thermal state on the orogeny style. Collision between two continents with hot **(a, b)** and cold **(c, d)** temperature structure of orogenic lithosphere. The models are all equipped with lithospheric mantle depletion density of -30 kg/m^3, as well as mafic continental lower crust and felsic orogenic lower crust. The simulation results are presented at times of 12 Myr and 25 Myr after model initiation. For the hot orogen **(a, b)**, the temperature at the Moho (700 °C) is 100 °C hotter than that of the continents (600 °C). For the cold orogen **(c, d)**, the temperature at the Moho (600 °C) is same to that of the continents (600 °C). The initial temperature increases linearly from 0 °C at the model surface to 600 °C or 700 °C at the crust base, and continues to increase to 1330 °C at the lithosphere base. Convergence rate is 10 mm/yr. Other parameters contrast between the orogen and its bounding continents are presented in Tables S1 and S2.

Supplementary Information References:

1. Xu, X. et al. Permian plume-strengthened Tarim lithosphere controls the Cenozoic deformation pattern of the HimalayanTibetan orogen. *Geology* **49**, 96–100 (2021).
2. Xiong, S., Tong, J., Ding, Y. & Li, Z. Aeromagnetic data and geological structure of continental China: A review. *Appl. Geophys.* **13**, 227–237 (2016).
3. Yin, H. & Zhou, J. *Magnetic Anomaly Map of China Mainland, Offshore and Adjacent Areas*. (Geological Publishing House, Beijing, 2018).
4. Yin, H., Zhou, J., Shu, Q. & Gao, W. The key technologies for making the magnetic anomaly map (1: 5,000,000) of China mainland, offshore and adjacent areas. *Prog. Geophys.* **30**, 2107–2112 (2015).
5. Xiong, S. et al. *Aeromagnetic Data and Geological Structure of Continental China*. (Geological Publishing House, Beijing, 2016).
6. Xiong, S. et al. *Aeromagnetic Series Map of China's Mainland and its Specification* 1: 2,500,000. (Geological Publishing House, 2013).
7. Yin, H. & Zhou, J. *Magnetic Anomaly Map of China Mainland, Offshore and Adjacent Areas*. (Geological Publishing House, Beijing, 2018).
8. Grant, F. S. Aeromagnetics, geology and ore environments, I. Magnetite in igneous, sedimentary and metamorphic rocks: an overview. *Geoexploration* **23**, 303–333 (1985).
9. Xiong, S., Tong, J., Ding, Y. & Li, Z. Aeromagnetic data and geological structure of continental China: A review. *Appl. Geophys.***13**, 227–237 (2016).
10. Telford, W. M., Geldart, L. P. & Sheriff, R. E. Applied geophysics. (Cambridge university press, 1990).
11. England, P. & Molnar, P. Rheology of the lithosphere beneath the central and western Tien Shan. *J. Geophys. Res. Solid Earth* **120**, 3803–3823 (2015).
12. Price, R. A. The Cordilleran foreland thrust and fold belt in the southern Canadian Rocky Mountains. *Geol. Soc. Lond. Spec. Publ.* **9**, 427–448 (1981).
13. Dong, S. et al. What drove continued continent-continent convergence after ocean closure? Insights from high-resolution seismic-reflection profiling across the Daba Shan in central China. *Geology* **41**, 671–674 (2013).
14. Maus, S. et al. EMAG2: A 2–arc min resolution Earth Magnetic Anomaly Grid compiled from

satellite, airborne, and marine magnetic measurements. *Geochem. Geophys. Geosyst.* **10**, https://doi.org/10.1029/2009GC002471 (2009).

15. Zheng, G. et al. Crustal deformation in the India-Eurasia collision zone from 25 years of GPS measurements. *J. Geophys. Res. Solid Earth* **122**, 9290–9312 (2017).
16. Zhang, B., Bao, X. & Xu, Y. Seismic anisotropy in the central Tien Shan unveils rheology-controlled deformation during intracontinental orogenesis. *Geology* **50**, 812–816 (2022).
17. Zhang, B., Bao, X. & Xu, Y. Distinct orogenic processes in the south-and north-central tien shan from receiver functions. *Geophys. Res. Lett.* **47**, e2019GL086941 (2020).
18. Li, W., Chen, Y., Yuan, X., Xiao, W. & Windley, B. F. Intracontinental deformation of the Tianshan Orogen in response to IndiaAsia collision. *Nat. Commun.* **13**, 3738 (2022).
19. Gerya, T. V. & Yuen, D. A. Characteristics-based marker-in-cell method with conservative finite-differences schemes for modeling geological flows with strongly variable transport properties. *Phys. Earth Planet. Inter.* **140**, 293–318 (2003).
20. Ranalli, G. *Rheology of the Earth*. (Springer Science & Business Media, 1995).
